# Supplementary material for: Molecular Mechanisms Associated with Clustered Lesion-Induced Impairment of 8-oxoG Recognition by the Human Glycosylase OGG1
Source: Molecules. 2021 Oct 26;26(21):6465. doi: 10.3390/molecules26216465 (PMC8587150; doi:10.3390/molecules26216465)
Supplement: Supplementary file 1 [file molecules-26-06465-s001.zip › SI/SI__Clustered_OGG1_Molecules.pdf]

Supplementary Materials: Molecular mechanisms associated with clustered lesion-induced impairment of 8-oxoG recognition by the human glycosylase OGG1

Tao Jiang [2], Antonio Monari [1,3], Elise Dumont [2,4] and Emmanuelle Bignon [1,*]

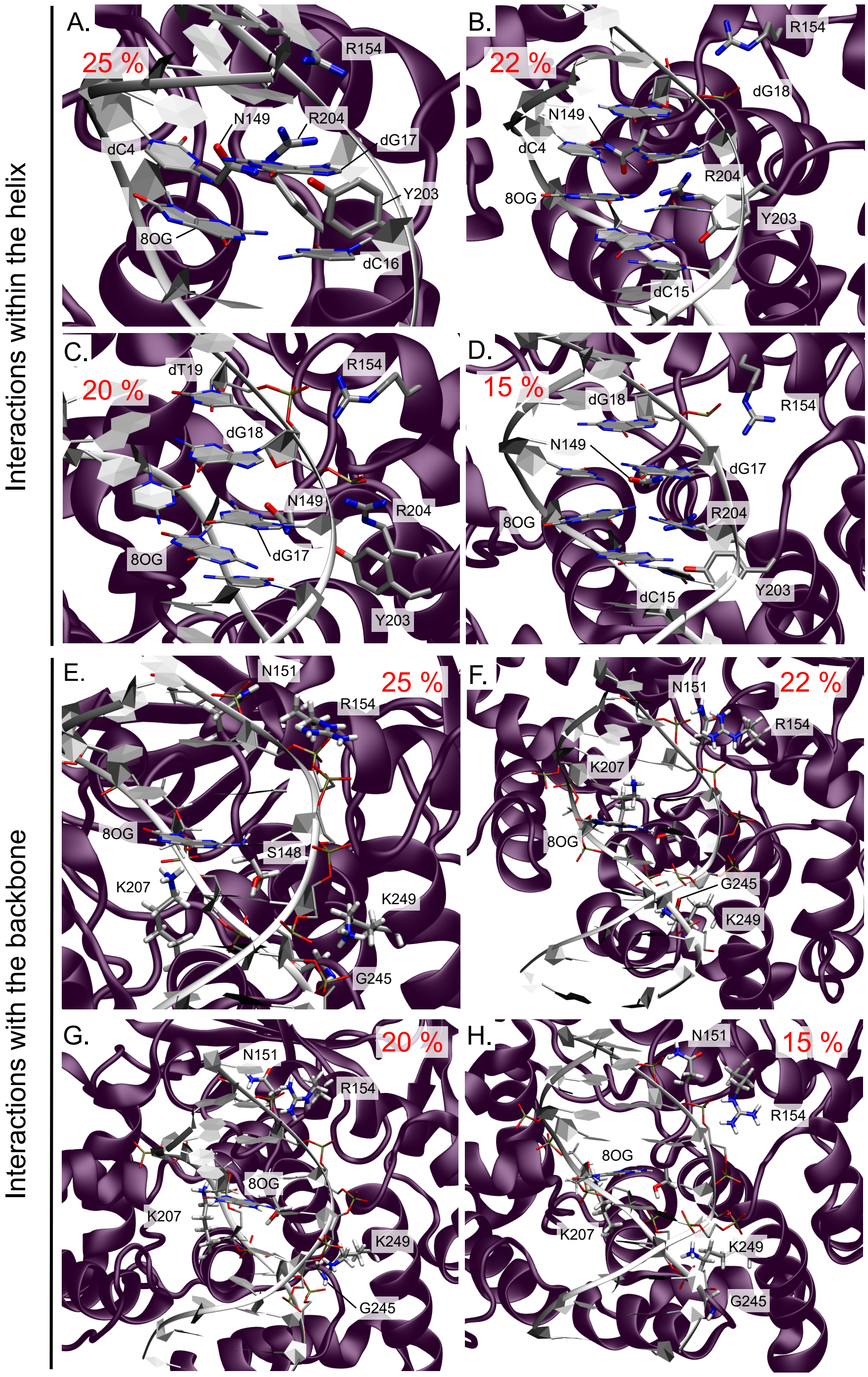

Figure S1. Representation of the key-interactions within the DNA helix (**A.** to **D.**) and with the DNA backbone (**E.** to **H.**) upon the presence of an isolated 8-oxoG for the four main clusters. The weight of the cluster is displayed in red.

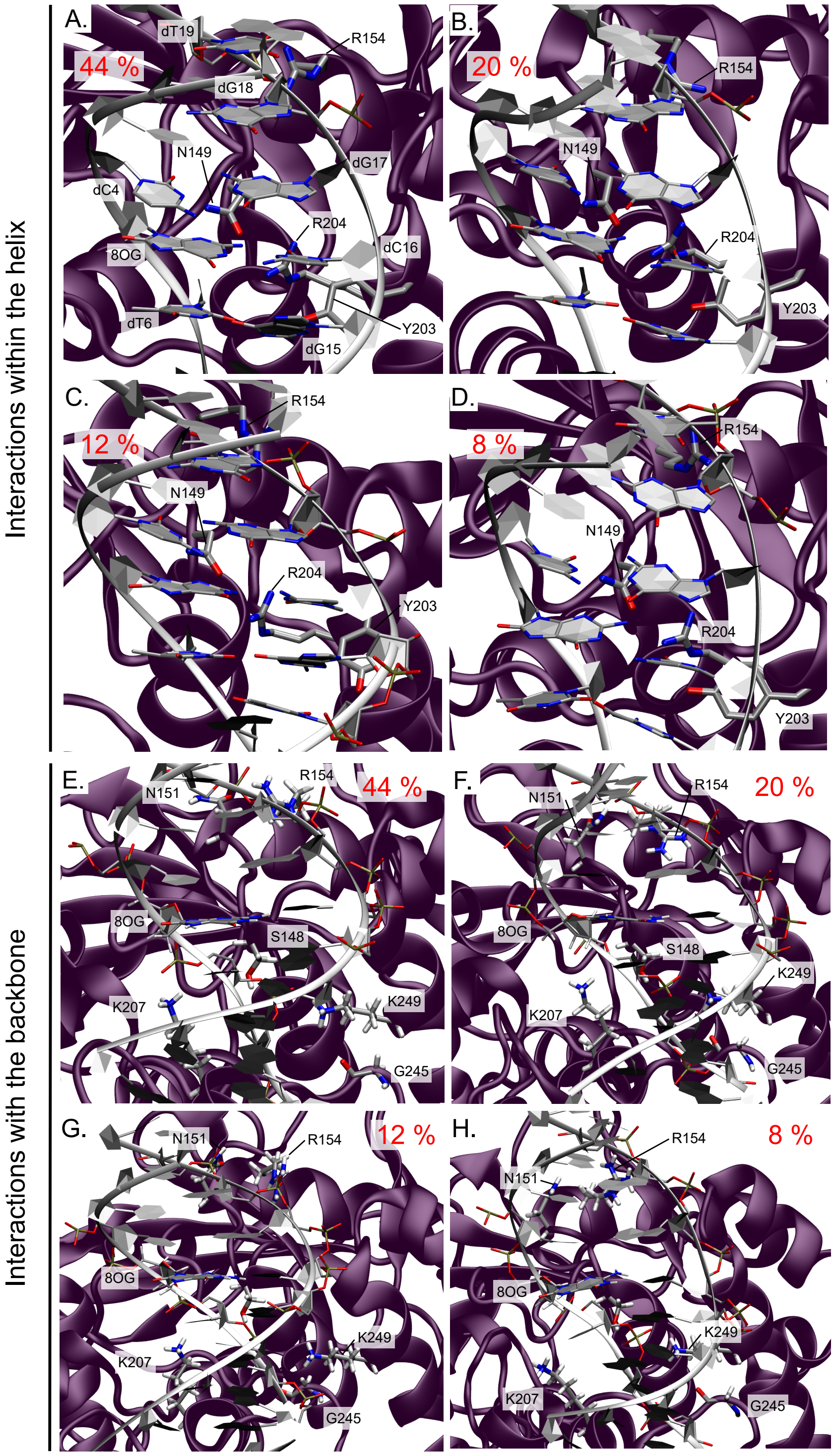

Figure S2. Representation of the key-interactions within the DNA helix (**A.** to **D.**) and with the DNA backbone (**E.** to **H.**) upon the presence of clustered 8-oxoG and 3′ mismatch for the four main clusters. The weight of the cluster is displayed in red.

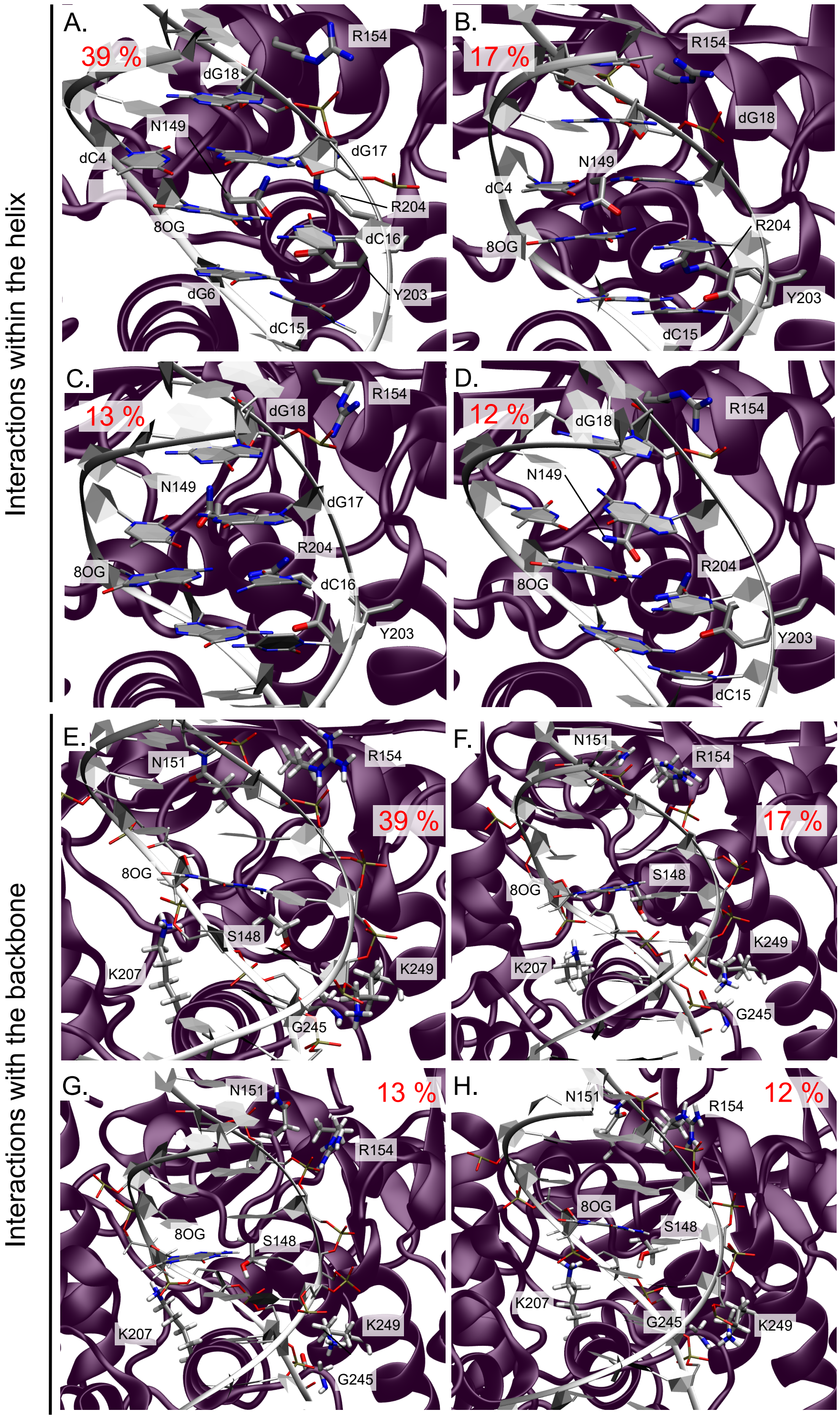

Figure S3. Representation of the key-interactions within the DNA helix (**A.** to **D.**) and with the DNA backbone (**E.** to **H.**) upon the presence of clustered 8-oxoG and 5′ mismatch for the four main clusters. The weight of the cluster is displayed in red.

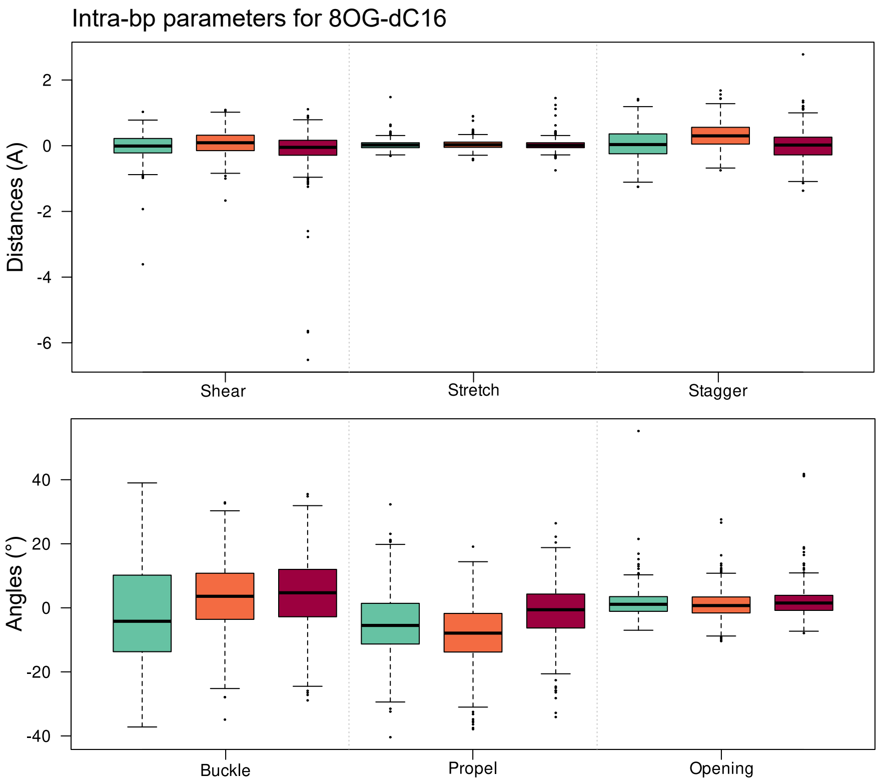

Figure S4. Intra-base pair structural parameters of the 8-oxoG-dC16 base pair in systems harboring an isolated 8-oxoG (cyan), clustered 8-oxoG + 3′ mismatch (orange) or clustered 8-oxoG + 5′ mismatch (red).

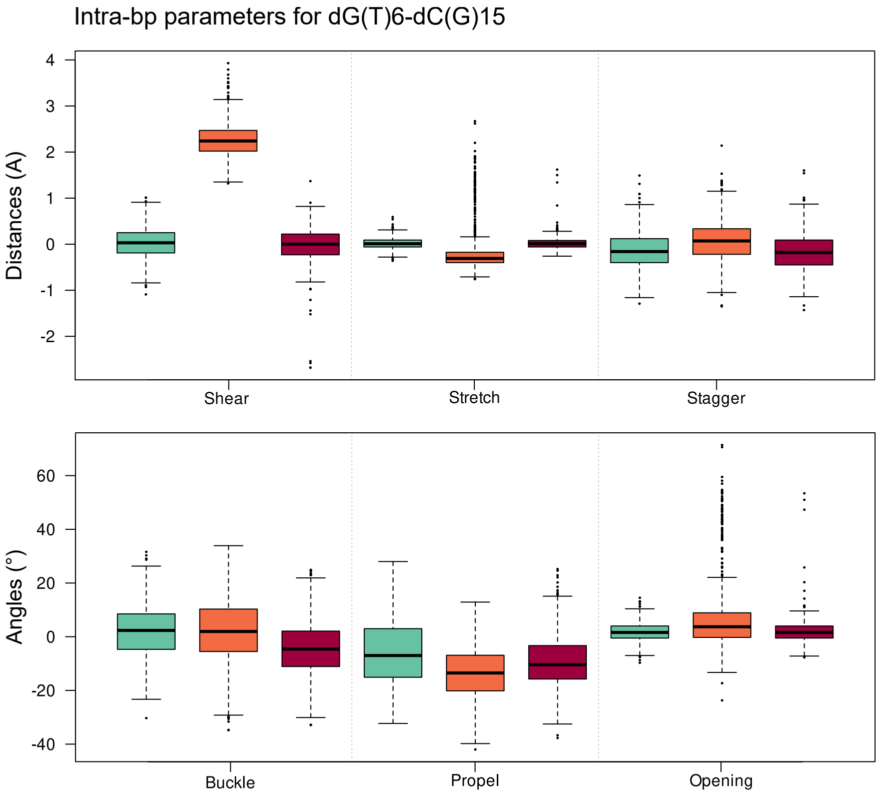

Figure S5. Intra-base pair structural parameters of the dG(T)6-dC(G)15 base pair 3′ to the 8-oxoG in systems harboring an isolated 8-oxoG (cyan), clustered 8-oxoG + 3′ mismatch (orange) or clustered 8-oxoG + 5′ mismatch (red).

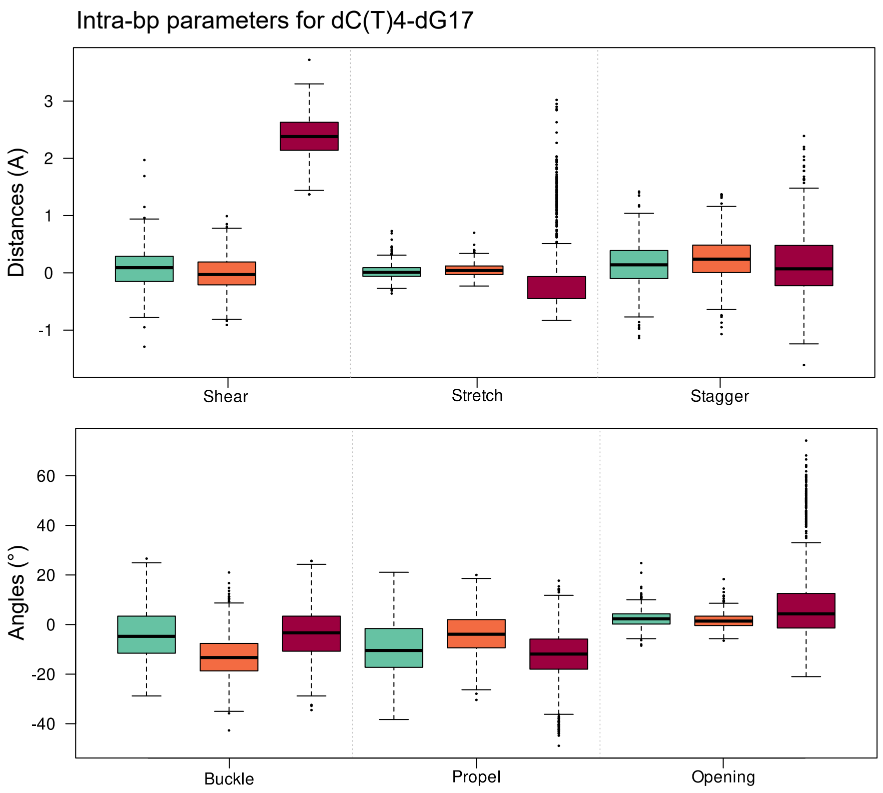

Figure S6. Intra-base pair structural parameters of the dC(T)4-dG17 base pair 3′ to the 8-oxoG in systems harboring an isolated 8-oxoG (cyan), clustered 8-oxoG + 3′ mismatch (orange) or clustered 8-oxoG + 5′ mismatch (red).

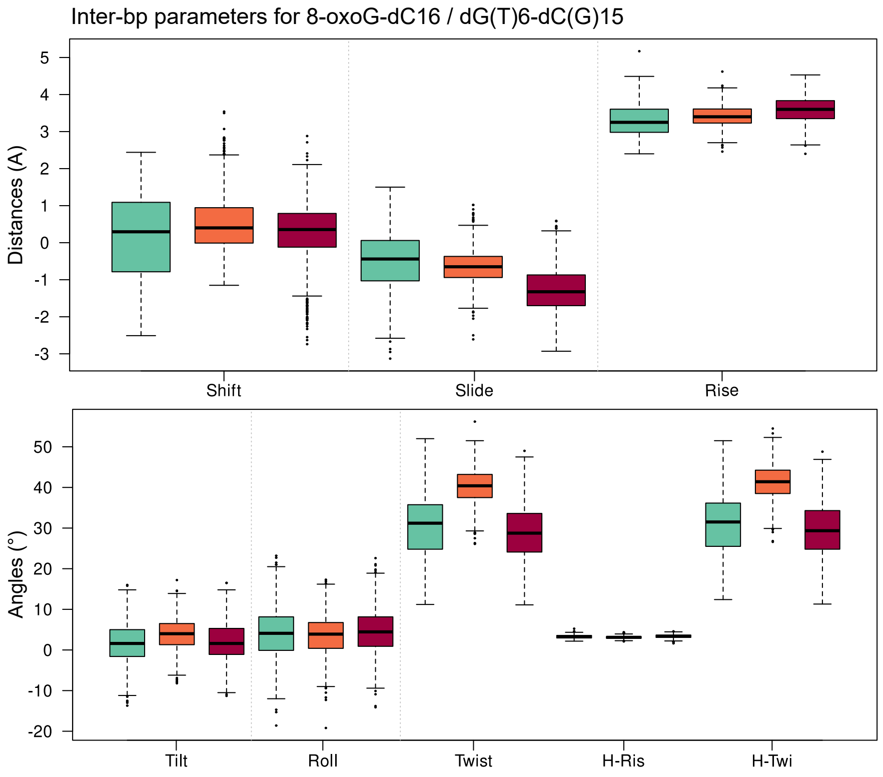

Figure S7. Inter-base pairs structural parameters of the 8-oxoG-dC16 / dG(T)6-dC(G)15 base pairs in systems harboring an isolated 8-oxoG (cyan), clustered 8-oxoG + 3′ mismatch (orange) or clustered 8-oxoG + 5′ mismatch (red).

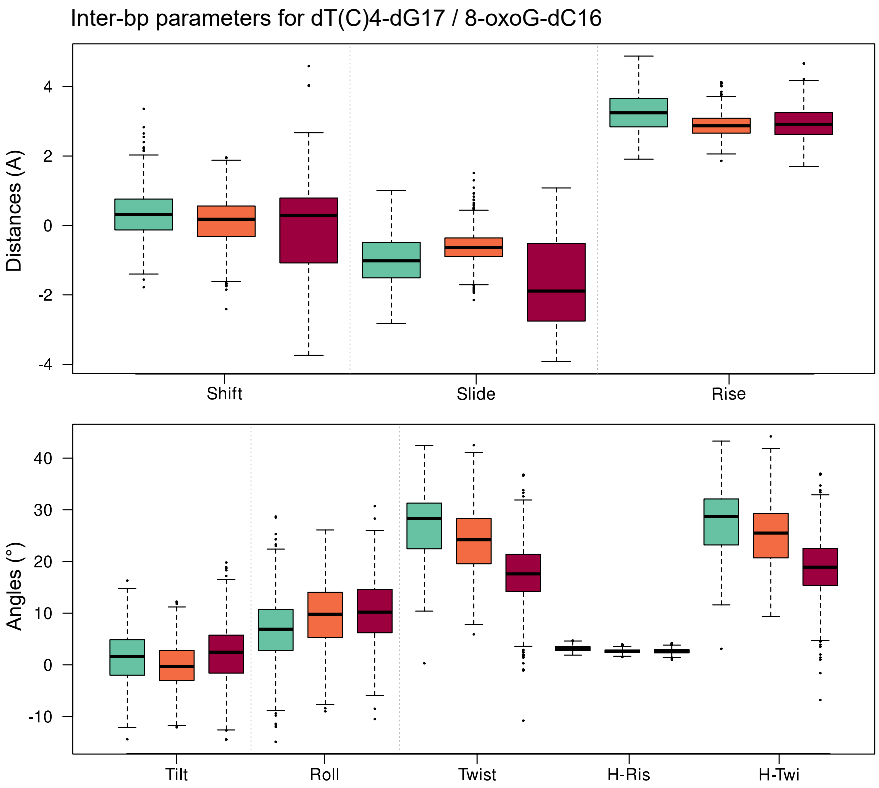

Figure S8. Inter-base pairs structural parameters of the dC(T)4-dG17 / 8-oxoG-dC16 base pairs in systems harboring an isolated 8-oxoG (cyan), clustered 8-oxoG + 3′ mismatch (orange) or clustered 8-oxoG + 5′ mismatch (red).

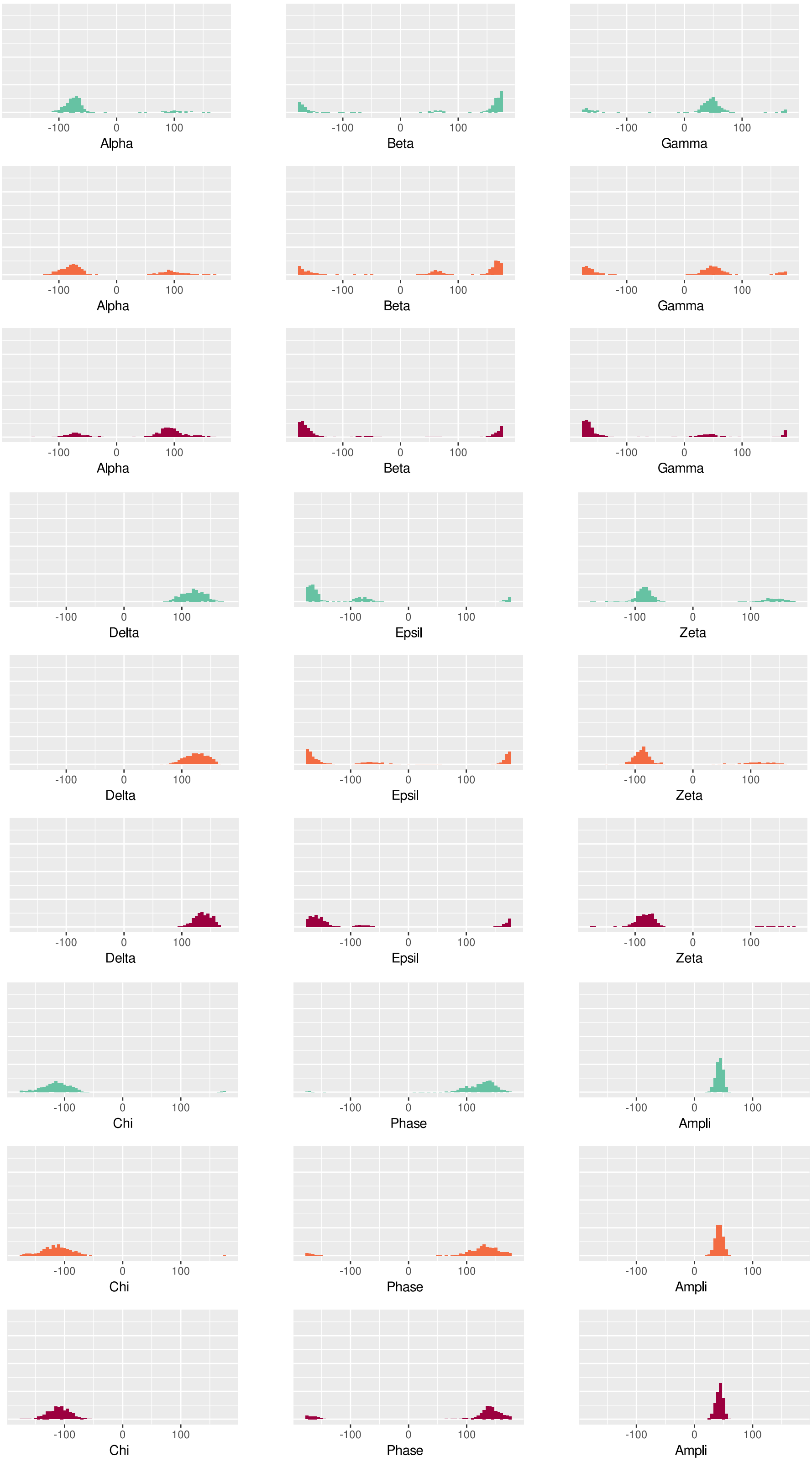

Figure S9. Distribution of 8-oxoG backbone angles values in systems harboring an isolated 8-oxoG (cyan), clustered 8-oxoG + 3′ mismatch (orange) or clustered 8-oxoG + 5′ mismatch (red).

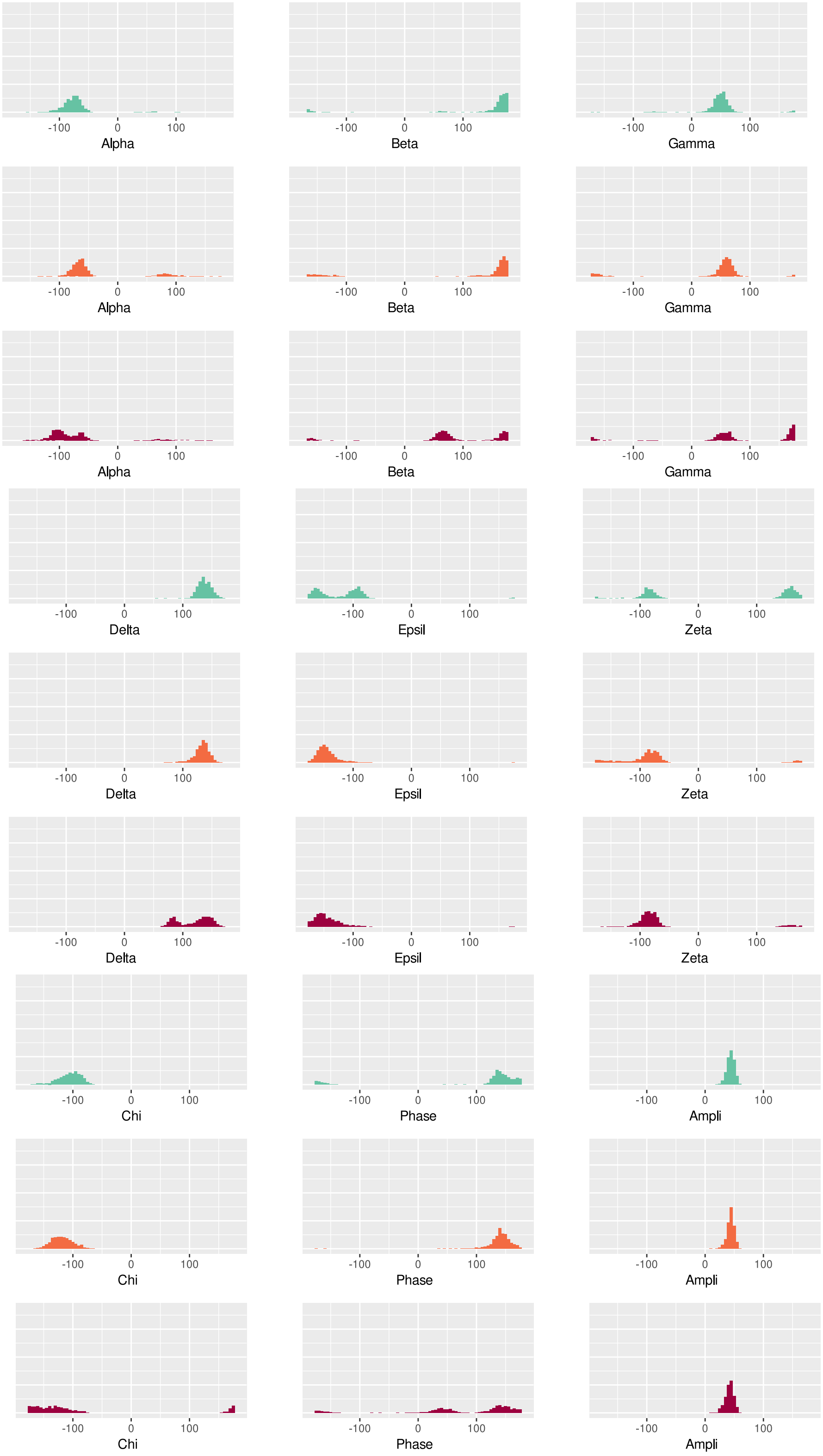

Figure S10. Distribution of dG(T)6 backbone angles values in systems harboring an isolated 8-oxoG (cyan), clustered 8-oxoG + 3′ mismatch (orange) or clustered 8-oxoG + 5′ mismatch (red).

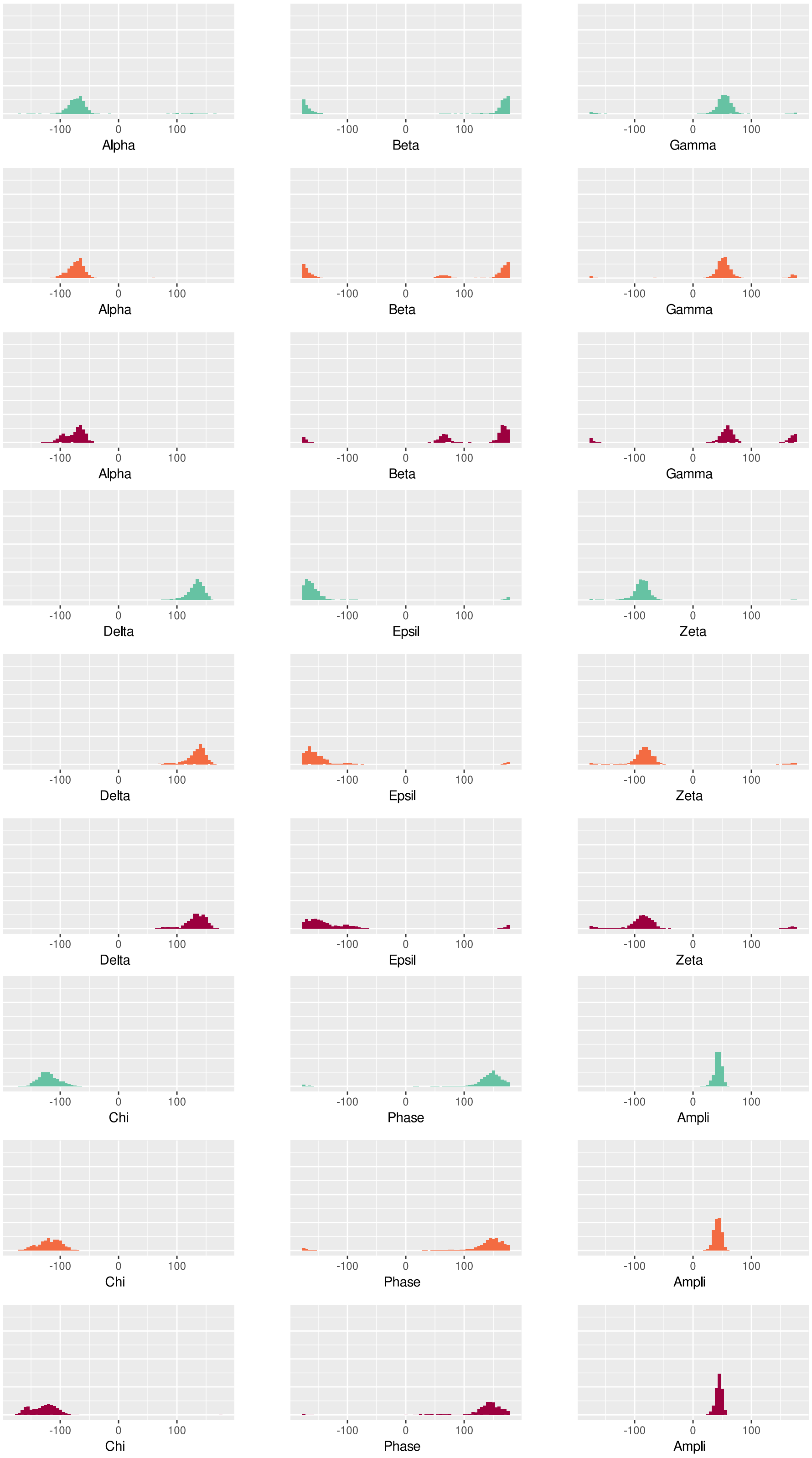

Figure S11. Distribution of the dC(T)4 backbone angles values in systems harboring an isolated 8-oxoG (cyan), clustered 8-oxoG + 3′ mismatch (orange) or clustered 8-oxoG + 5′ mismatch (red).
